# RuBisCO-based CO_2_ fixation improves glutamate production in *Corynebacterium glutamicum*

**DOI:** 10.3389/fbioe.2026.1783749

**Published:** 2026-02-27

**Authors:** Aiying Wei, Jingui Liu, Yulin Tang, Gang Meng, Chunguang Zhao, Houbo Su, Heyun Wu, Qian Ma, Xixian Xie

**Affiliations:** 1 College of Biotechnology, Tianjin University of Science and Technology, Tianjin, China; 2 Ningxia Eppen Biotech Co., Ltd., Yinchuan, China; 3 Key Laboratory of Industrial Fermentation Microbiology, Ministry of Education, Tianjin University of Science and Technology, Tianjin, China

**Keywords:** Calvin-cycle, *Corynebacterium glutamicum*, glutamate, metabolic engineering, RuBisCO

## Abstract

**Background and introduction:**

Efficiently harnessing CO_2_ for the bioproduction of chemicals stands as an important way to mitigate CO_2_ emissions and actively advance the achievement of carbon neutrality. Drawing inspiration from the natural Calvin-Benson-Bassham (CBB) cycle for CO_2_ fixation, the heterologous introduction of phosphoribulokinase (PRK) and ribulose-1,5-bisphosphate carboxylase/oxygenase (RuBisCO) into microbial cell factories emerges as a highly promising method for fully harnessing CO_2_ for bioproduction purposes.

**Methods:**

In this study, we engineered the industrial glutamate-hyperproducing strain *Corynebacterium glutamicum* YPGlu001 by introducing a heterologous RuBisCO-PRK pathway. Two metabolic configurations were evaluated: a “replacement” strategy, which blocked native glycolytic and pentose phosphate pathway (PPP) fluxes (via Δ*gap*, Δ*gapX*, Δ*pgk*, and Δ*zwf*) to force carbon through the CBB shunt; and a “complementation” strategy, where the CO_2_-fixation pathway supplemented the native central metabolism. Pathway performance was optimized through promoter engineering (P_tac_, P_H30_, P_fba_, P_groES_) and adaptive laboratory evolution (ALE) under increasing CO_2_ stress.

**Results:**

Comparative analysis revealed that the “replacement” strategy severely impaired cell growth and glutamate synthesis, with ALE failing to restore the desired production levels. In contrast, the “complementation” strategy significantly enhanced metabolic performance. The optimized strain GluE014 exhibited superior carbon-to-product conversion, achieving a glutamate titer of 196.78 g/L in a 5 L fed-batch fermenter within 30 h. This represents a 13.94% increase in titer and an 11.55% improvement in glucose-based yield compared to the parental strain. Furthermore, the engineered strain demonstrated improved carbon economy, reducing glucose consumption by 5.24% while maintaining high productivity.

**Conclusion:**

This work demonstrates that “complementing” native metabolism with a CO_2_-fixation shunt is more effective than “replacing” essential pathways in industrial *C. glutamicum*. By successfully integrating heterologous CO_2_ assimilation with robust industrial fermentation, this study provides a scalable and efficient blueprint for developing next-generation, carbon-negative microbial cell factories.

## 1 Introduction

Global warming has emerged as a critical issue that profoundly impacts the destiny of every individual on the Earth, and it is intricately linked to the emission of CO_2_. To address this issue, many countries globally have collaborated to implement measures aimed at reducing CO_2_ emissions. Nevertheless, it is crucial to acknowledge that CO_2_ serves as a vital carbon source for a diverse array of organisms on the Earth, including plants, cyanobacteria, and various prokaryotic microbes (Lin et al., 2019). It has been reported that nearly 100 gigatons of carbon can be fixed via plants, algae, and photosynthetic bacteria (Prywes et al., 2025). Drawing inspiration from nature, the utilization of CO_2_ for the bioproduction of chemicals through microbial cell factories has emerged as a highly promising strategy in the synthetic biology era (Zhao et al., 2024), offering a pathway to transform excess CO_2_ into valuable products (Xia et al., 2017; Hu et al., 2018; Tseng et al., 2018).

The Calvin-Benson-Bassham (CBB) cycle is responsible for 90% of carbon fixation and stands as the preeminent pathway for carbon assimilation (Zhou et al., 2025). A pivotal enzyme in this process is ribulose-1,5-bisphosphate carboxylase/oxygenase (RuBisCO), which plays a crucial role in facilitating CO_2_ fixation through the CBB cycle in both plants and photosynthetic microbes. Additionally, another key enzyme, phosphoribulokinase (PRK), is essential as it supplies the substrate ribulose-1,5-bisphosphate (RuBP), enabling RuBisCO to carry out CO_2_ fixation effectively. Except for PRK and RuBisCO, the remaining enzymes involved in the CBB cycle are universally found in the majority of microbes that engage in glycolysis and the pentose phosphate pathway (PPP). On basis of above facts, a heterologous PRK-RuBisCO shunt has been incorporated into organisms such as *Saccharomyces cerevisiae* (Guadalupe-Medina et al., 2013) and *Escherichia coli* (Ting and Ng, 2023; Zhou et al., 2025), enabling CO_2_ fixation and promoting the synthesis of targeted products.

*Corynebacterium glutamicum* is an important industrial microorganism that has been widely used for the production of various amino acids, with a particular emphasis on the bulk amino acid, glutamate. The fermentation of *C. glutamicum* has achieved a glutamate titer exceeding 200 g/L; however, the yield of glutamate from glucose has emerged as a primary constraint influencing production costs. Although the theoretical yield of glutamate from glucose stands at 81.7%, industrial production typically achieves only around 69%–71% due to carbon losses incurred through CO_2_ emissions. Implementing CO_2_ fixation within *C. glutamicum* could significantly enhance glutamate yields (Chinen et al., 2007). As a widely utilized amino acid, a slight increase in yield would substantially impact the production costs and the overall economic profitability. The heterologous expression of RuBisCO has been attempted in *C. glutamicum* (Baumgart et al., 2017), yet, the effect of the PRK-RuBisCO shunt on glutamate synthesis has not been extensively explored.

In this study, the *cbbL* and *cbbS* genes encoding two subunits of the RuBisCO protein from *Halothiobacillus neapolitanus* and the *prk* gene from *Methanospirillum hungatei* were introduced into the industrial glutamate producing strain. The replacement of the RuBisCO pathway with glycolysis and PPP was established, yet the efficiency for glutamate production is far from satisfactory. In contrast, the complementation of the RuBisCO pathway with the central carbon metabolism successfully improved the titer and yield of glutamate in the engineered strain, with glutamate production rising from 172.71 g/L to 196.78 g/L with an increase of 13.94%, and the yield increased from 63.23% to 70.53% with an increase of 11.55%. The above results suggested the potential of integrating CO_2_ fixation into industrial bioproduction as a highly efficient strategy for the synthesis of valuable chemicals.

## 2 Materials and methods

### 2.1 Bacterial strains, plasmids and chemicals

The strains and plasmids used in this study are listed in Table 1, and the primers used in this study are listed in Supplementary Table S1. *E. coli* DH5α was used as the cloning host for plasmid construction, and *C. glutamicum* YPGlu001 was used as the starting strain for genomic manipulations. Plasmid pK18mobsacB was applied for genomic manipulations. The plasmids pEC-XK99E and pZ8 were used as the vectors for gene overexpression. The chemicals and enzymes for gene manipulation were obtained from Takara and NEB. The carbonic anhydrase and creatine-phosphate kinase for the carry out of enzymatic activity assay were purchased from Sigma-Aldrich.

**TABLE 1:** Strains and plasmids used in this study.

Strains/plasmids	Characteristics	Source
Strains		
*E. coli* DH5α	Cloning host	Lab stock
*C. glutamicum*	Wild type	Lab stock
ATCC13869		
YPGlu001	Glutamate high-yielding strain	Lab stock
GluE001	YPGlu001, Δ*gap*	This study
GluE002	GluE001, Δ*gapX*	This study
GluE003	YPGlu001, Δ*pgk*	This study
GluE004	GluE002, Δ*zwf*	This study
GluE005	GluE003, Δ*zwf*	This study
GluE006	GluE004, Δ(BBD29_05350-BBD29_05355):: (P_tac_-*cbbLS*)	This study
GluE007	GluE005, Δ(BBD29_05350-BBD29_05355):: (P_tac_-*cbbLS*)	This study
GluE008	GluE004, Δ(BBD29_05350-BBD29_05355):: (P_H30_-*cbbLS*)	This study
GluE009	GluE005, Δ(BBD29_05350-BBD29_05355):: (P_H30_-*cbbLS*)	This study
GluE010	GluE006, Δ(BBD29_04380-BBD29_04385):: (P_fba_-*prk*)	This study
GluE011	GluE006, Δ(BBD29_04380-BBD29_04385):: (P_groES_-*prk*)	This study
GluE012	GluE007, Δ(BBD29_04380-BBD29_04385):: (P_fba_-*prk*)	This study
GluE013	GluE007, Δ(BBD29_04380-BBD29_04385):: (P_groES_-*prk*)	This study
GluE014	YPGlu001, Δ(BBD29_05350-BBD29_05355):: (P_tac_-*cbbLS*), Δ(BBD29_04380-BBD29_04385):: (P_groes_-*prk*)	This study
GluE015	YPGlu001, Δ(BBD29_05350-BBD29_05355):: (P_tac_-*cbbLS*), Δ(BBD29_04380-BBD29_04385):: (P_fba_-*prk*)	This study
GluP001	YPGlu001, pXMJ19-P_tac_-*cbbLS*	This study
GluP002	YPGlu001, pXMJ19-P_tuf_-*cbbLS*	This study
GluP003	YPGlu001, pXMJ19-P_pgk_-*cbbLS*	This study
GluP004	YPGlu001, pXMJ19-P_H5_-*cbbLS*	This study
GluP005	YPGlu001, pXMJ19-P_H30_-*cbbLS*	This study
GluP006	YPGlu001, pXMJ19-P_H36_-*cbbLS*	This study
GluP007	GluP001, pZ8-P_tac_-*prk*	This study
GluP008	GluP001, pZ8-P_sod_-*prk*	This study
GluP009	GluP001, pZ8-P_fba_-*prk*	This study
GluP010	GluP001, pZ8-P_groES_-*prk*	This study
GluP011	GluP005, pZ8-P_tac_-*prk*	This study
GluP012	GluP005, pZ8-P_sod_-*prk*	This study
GluP013	GluP005, pZ8-P_fba_-*prk*	This study
GluP014	GluP005, pZ8-P_groES_-*prk*	This study
Plasmids		
pK18mobsacB	kan^r^, shuttle vector	Lab stock
pEC-XK99E	kan^r^, trc promoter, expression vector	Lab stock
pXMJ19	Cm^r,^ expression vector	Lab stock
pZ8	Kan^r^, expression vector	Lab stock
pXMJ19-P_tac_-*cbbLS*	Cm^r^, P_tac_-*cbbLS* expression vector	This study
pXMJ19-P_tuf_-*cbbLS*	Cm^r^, P_tuf_-*cbbLS* expression vector	This study
pXMJ19-P_pgk_-*cbbLS*	Cm^r^, P_pgk_-*cbbLS* expression vector	This study
pXMJ19-P_H5_-*cbbLS*	Cm^r^, P_H5_-*cbbLS* expression vector	This study
pXMJ19-P_H30_-*cbbLS*	Cm^r^, P_H30_-*cbbLS* expression vector	This study
pXMJ19-P_H36_-*cbbLS*	Cm^r^, P_H36_-*cbbLS* expression vector	This study
pZ8-P_tac_-*prk*	Kan^r^, P_tac_-*prk* expression vector	This study
pZ8-P_sod_-*prk*	Kan^r^, P_sod_-*prk* expression vector	This study
pZ8-P_fba_-*prk*	Kan^r^, P_fba_-*prk* expression vector	This study
pZ8-P_groES_-*prk*	Kan^r^, P_groES_-*prk* expression vector	This study

### 2.2 Chromosomal knockout of genes in *Corynebacterium glutamicum*

For the knockout of *gap*, *gapX* and *zwf* genes in *C. glutamicum*, homologous recombination based on plasmid pK18mobsacB was applied. The up/down-stream homologous arms amplified from the genome of *C. glutamicum* YPGlu001, were assembled with amplified pK18mobsacB by NEBuilder® HiFi DNA Assembly Cloning Kit. The recombinant plasmids pK18mobsacB-Δ*gap*, pK18mobsacB-Δ*gapX*, and pK18mobsacB-Δ*zwf* were respectively constructed.

Cells were inoculated and precultured overnight in 50 mL YPG medium (30 g/L glucose, 12 g/L yeast extract, 17 g/L soybean meal powder, 10 g/L urea, 0.4 g/L MgSO_4_, 1 g/L succinic acid, 300 μg/L V_H_, 200 μg/L V_B1_) in a 500 mL Erlenmeyer flask at 200 rpm, 30 °C. Then, inoculate 2 mL of the preculture to 50 mL YPG-BHES medium containing 1.85% brain heart extract and 9.1% sorbitol in a 500 mL Erlenmeyer flask. Cells were harvested for the preparation of electrocompetent cells, when the cell OD_600_ reached around 1.75 after culturing for about 2–4 h. The electroporation was performed at 2200 V, and the resulting pulse duration were about 3.5–4.5 ms. After electroporation, cells were transferred immediately into 0.5 mL prewarmed YPG-BHES medium and incubated for exactly 6 min at 46 °C. After the heat shock, allow cells to regenerate with shaking for 1 h at 37 °C and another 1 h at 30 °C. Plate the cells on YPG-BHES plates containing 50 μg/mL kanamycin, and incubate the plates at 30 °C for 2 days. Then, the positive colonies grown on the plate were selected and the successful single crossover homologous recombination was verified by PCR. The correct colonies were then plated on YPG-Suc medium plates containing 15% sucrose and cultured at 30 °C for 2 days. The colonies that grown on the YPG-Suc medium plates were respectively plated on YPG plates with and without 50 μg/mL kanamycin. The positive colonies that could grow on YPG plate without kanamycin, but could not grow on YPG plate with kanamycin were selected and further verified via PCR and DNA sequencing.

### 2.3 Enzymatic activity assay

Glyceraldehyde-3-phosphate dehydrogenase (GAPDH) activity was measured using a commercial GAPDH Activity Assay Kit (Solarbio, China) following the manufacturer’s protocol. Briefly, bacterial cells were cultured in BHES medium to the exponential phase (3–5 h) and harvested by centrifugation. The cell pellets were resuspended in extraction buffer and disrupted by ultrasonication. After centrifugation (10,000 rpm, 20 min, 4 °C), the supernatant was collected as the crude enzyme extract. GAPDH activity was determined based on the conversion of 1,3-bisphosphoglycerate and NADH to 3-phosphoglycerate, inorganic phosphate, and NAD^+^, with the decrease in NADH absorbance at 340 nm used to quantify enzyme activity.

Phosphoglycerate kinase (PGK) activity was determined using a PGK Activity Assay Kit (Solarbio, China) according to the manufacturer’s instructions. The preparation of the crude enzyme extract was performed as described for the GAPDH assay. PGK catalyzes the conversion of 3-phosphoglycerate and ATP into 1,3-bisphosphoglycerate and ADP, which is subsequently coupled to the oxidation of NADH to NAD^+^ in a secondary reaction catalyzed by glyceraldehyde-3-phosphate dehydrogenase. The decrease in NADH absorbance at 340 nm was used to quantify PGK activity.

RuBisCO activity was determined using a RuBisCO Activity Assay Kit (Solarbio, China) according to the manufacturer’s instructions. Bacterial cells were cultured in 50 mL of CGXII medium for 24 h, harvested by centrifugation, and resuspended in RuBisCO extraction buffer. The cells were disrupted by vortexing with glass beads, and the lysate was centrifuged at 12,000 g for 10 min at 4 °C to obtain the crude enzyme extract. RuBisCO catalyzes the carboxylation of ribulose-1,5-bisphosphate (RuBP) to generate 3-phosphoglycerate (3-PGA), which is subsequently converted to glyceraldehyde-3-phosphate (GAP) by 3-phosphoglycerate kinase and glyceraldehyde-3-phosphate dehydrogenase, accompanied by the oxidation of NADH to NAD^+^. The decrease in absorbance at 340 nm over 5 min was used to quantify RuBisCO activity.

Phosphoribulokinase (PRK) activity was determined according to the method described by Tseng *et al.* (Tseng et al., 2018). The crude enzyme extract was prepared as described for RuBisCO. PRK catalyzes the phosphorylation of ribulose-5-phosphate to ribulose-1,5-bisphosphate with the concomitant conversion of ATP to ADP. In the coupled reaction system, pyruvate kinase converts phosphoenolpyruvate and ADP to pyruvate and ATP, and lactate dehydrogenase subsequently reduces pyruvate to lactate with the oxidation of NADH to NAD^+^. The decrease in NADH absorbance at 340 nm was used to quantify PRK activity.

### 2.4 Adaptive laboratory evolution

Cells were inoculated into 100 mL Erlenmeyer flasks containing 20 mL BHIS medium and cultivated overnight at 30 °C with shaking at 200 rpm. A 10 mL aliquot of the overnight culture was harvested by centrifugation (10 min, 3,200 × g, 4 °C) and washed with CGXII medium. The cell pellet was then resuspended and transferred into 500 mL flasks containing 100 mL CGXII medium, with the initial OD_600_ adjusted to 0.2. Cultures were incubated at 30 °C and 200 rpm in a 10% CO_2_-enriched incubator. Cell growth was monitored by periodically measuring OD_600_, examining cell morphology under a microscope, and verifying cell viability on BHIS agar plates. In the initial phase of serial cultivation passages, if no discernible growth was evident over a prolonged period, the culture was centrifugation every 2 weeks. The resulting cell pellet was then resuspended in freshly prepared, sterile CGXII medium and re-inoculated to mitigate the degradation of medium components. As the adaptive evolution process progressed, cell growth gradually improved, and once stable growth was established, samples were collected, and the culture was serially passaged upon reaching an OD_600_ of 3. Meanwhile, the residual cell culture from each passage was sustained for further cultivation to evaluate their growth plateau.

### 2.5 Shake-flask fermentation

A loop of cells grown on an agar slant was transferred to 50 mL seed medium in a 500 mL shake flask, which contained 30 g/L glucose, 12 g/L yeast extract, 17 g/L soybean meal powder, 10 g/L urea, 1 g/L succinic acid, 0.5 g/L methionine, 0.4 g/L MgSO_4_·7H_2_O, 10 mg/L FeSO_4_, 10 mg/L MnSO_4_, 0.1 mg/L V_B1_, and 0.2 mg/L V_H_. The seed cultivation lasted approximately 16 h at 30 °C with a shaking rate of 220 rpm. The seed culture was then transferred to a fresh fermentation medium containing 70 g/L glucose, 1.6 g/L KCl, 2.8 g/L KH_2_PO_4_, 1.85 g/L MgSO_4_·7H_2_O, 1.7 g/L aspartic acid, 1.5 g/L betaine, 10 mL/L molasses, 77 mL/L corn steep liquor, 0.6 mg/L V_B1_, 0.55 mg/L V_H_, 0.75 mg/L V_C_ and 4-aminobenzoic acid, 10 mg/L FeSO_4_ and MnSO_4_, 0.0025 mg/L V_B12_, 40 g/L CaCO_3_. Urea was used for adjustment of the pH around 7.0. Initially, a temperature of 30 °C and a shaking rate of 180 rpm were applied until 12 h of fermentation, after which time, the temperature was elevated to 37 °C and the shaking rate was increased to 220 rpm for another 36 h.

### 2.6 Fed-batch fermentation in a 5 L bioreactor

Fed-batch fermentation was conducted in a 5 L bioreactor (BIOTECH-5BG, BaoXing Bio, Shanghai, China). The seed medium was the same as used in the shake-flask fermentation. The seed culture was first grown in a bioreactor with a working volume of 3 L. The dissolved oxygen (DO) was maintained at about 25% by adjusting the stirring speed and the aeration rate. The pH and temperature were controlled at 7.0 °C and 30 °C, respectively. When the OD_600_ reached 10, the culture was transferred to the fermenter for further fermentation.

The fermentation medium contained 40 g/L glucose, 0.8 g/L phosphoric acid, 2.2 g/L potassium chloride, 120 g/L corn syrup, 1 g/L soybean meal extract, 1.4 g/L MgSO_4_·7H_2_O, 8.0 mg/L FeSO_4_·7H_2_O, 1.0 mg/L MnSO_4_·H_2_O, 0.6 g/L V_H_, 0.05 mg/L V_B1_, and a 1 mL/L V_C_. The loading volume was 3 L, with an inoculation ratio of 15%. The pH was kept at 7.0 by the addition of ammonia, and the temperature was initially set at 32 °C, which was gradually elevated to 38.5 °C to facilitate the production and secretion of glutamate. The pH and DO were set at 7.0% and 20%–50%, respectively. Glucose solution with a concentration of 65% was added at an appropriate rate to keep the concentration of glucose in the medium within 5 g/L.

### 2.7 Analytical methods

Glutamate in the fermentation broth was analyzed via HPLC (Agilent-1260), using an Agilent ZORBAX Eclipse AAA 4.6 × 150 mm refractive index detector. KH_2_PO_4_ solution (5.44 g/L, pH7.2) was used as the eluent with a flow rate of 1 mL/min at 40 °C.

## 3 Results

### 3.1 Block of glycolysis and PPP pathway in *Corynebacterium glutamicum*

**FIGURE 1:**
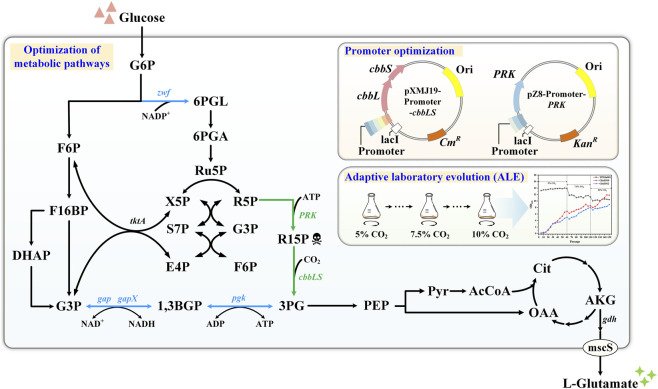
Engineering strategy for efficient glutamate production in *C. glutamicum* aided by heterologous PRK–RuBisCO pathway. Optimization of metabolic pathways: Blue lines denote targeted gene knockouts in two distinct combinations (i) Δ*gap*, Δ*gapX* and Δ*zwf* (ii) Δ*pgk* and Δ*zwf*. Green lines indicate introduction of the heterologous RuBisCO pathway. Promoter optimization: Plasmid-based screening was conducted to identify optimal promoters driving *cbbLS* and *prk* expression. Adaptive laboratory evolution (ALE): Engineered strains were serially cultured under varying CO_2_ concentrations to restore growth and enhance key enzyme activities.

In this study, we aim to delineate the heterologous RuBisCO pathway for CO_2_ fixation within the glutamate-overproducing strain *C. glutamicum* YPGlu001. To achieve this goal, we first substituted the RuBisCO pathway for glycolysis and the PPP pathway (as illustrated in Figure 1). Given that the integration of the RuBisCO pathway could potentially replenish the intermediate 3-phosphoglycerate (3-PG) in the glycolytic pathway, we sought to create a metabolic drive for the RuBisCO pathway by inhibiting the endogenous biosynthesis of 3-PG from upstream glycolysis. Consequently, we sequentially disrupted the *gap* and *gapX* genes, which encode glyceraldehyde-3-phosphate dehydrogenase (GAPDH), to block the conversion from glyceraldehyde-3-phosphate (G3P) to 1, 3-bisphospho-glycerate (1, 3-BGP) in *C. glutamicum* YPGlu001, resulting in the construction of strains GluE001 and GluE002, respectively. Furthermore, the knockout of the *pgk* gene, which encodes the phosphoglycerate kinase (PGK), would effectively halt the conversion of the precursor 1, 3-BGP into 3-PG. Consequently, to facilitate a comparative analysis with the disruptions of the *gap* and *gapX* genes, we deleted the *pgk* gene in *C. glutamicum* YPGlu001, resulting in the creation of strain GluE003.

**FIGURE 2:**
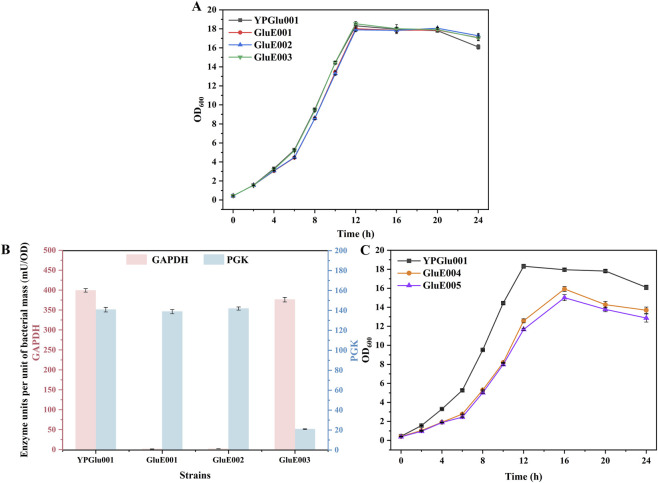
Effects of disrupting glycolytic and pentose phosphate pathways on strain growth. **(A)** Growth curves of YPGlu001, GluE001, GluE002, and GluE003 strains cultured in shake flasks for 24 h. **(B)** GAPDH and PGK enzyme activities in YPGlu001, GluE001, GluE002, and GluE003 strains. **(C)** Growth curves of YPGlu001, GluE004, and GluE005 strains cultured in shake flasks for 24 h. Data points in A to C are presented as mean ± SD from three independent biological replicates.

The growth profiles of strains GluE001, GluE002, and GluE003 were compared with that of the parental strain, which served as the control, and the results are illustrated in Figure 2A. As depicted in the figure, the growth of GluE001, GluE002 and GluE003 was not affected. Furthermore, to conclusively verify the loss of function in GAPDH and PGK, enzymatic assays for GAPDH and PGK were performed on GluE001, GluE002, and GluE003, respectively. As demonstrated in Figure 2B, the enzymatic activity of GAPDH in GluE001 and GluE002 was negligible when compared to the control strain *C. glutamicum* YPGlu001, signifying the complete inactivation of GAPDH. In addition, the enzymatic activity of PGK in GluE003 was neglectable.

To further impede the PPP metabolism, we sequentially knocked out the *zwf* gene in strains GluE002 and GluE003, thereby generating strains GluE004 and GluE005, respectively. We subsequently evaluated the cell growth of GluE004 and GluE005 in shake-flask fermentation, and the results are presented in Figure 2C. Compared to strain *C. glutamicum* YPGlu001, the cell growth of strain GluE004 exhibited a marked slowdown, with the cell OD_600_ reaching a plateau value of 15.92, which was 11.31% lower than that of strain YPGlu001. Similarly, strain GluE005 showed a comparable reduction in growth relative to YPGlu001, with an OD_600_ plateau of 15.01 (16.38% lower).

Theoretically, blocking the main glycolytic pathway (Δ*gap* and Δ*pgk*) and the pentose phosphate pathway (Δ*zwf*) should severely impair growth when glucose is the sole carbon source. However, strains GluE004 and GluE005 did maintain relatively normal growth without the expected severe impairment. To further confirm the successful deletion of these genes, we measured the relative transcription levels of these genes with their expression in *C. glutamicum* ATCC13869 as the control, and the results are shown in Supplementary Figure S2. The transcription levels of the deleted genes in GluE004 and GluE005 were virtually undetectable, confirming that the genes were successfully knocked out and ruling out incomplete deletion. It is probably that the addition of yeast extract in the fermentation medium provides essential nutrients, such as amino acids and organic acids, which can bypass the blocked glycolytic steps and support growth. Considering that strains GluE004 and GluE005 exhibit similar growth characteristics, both strains were subjected to subsequent modifications simultaneously.

### 3.2 Introduction of heterologous RuBisCO-based CO_2_ fixation pathway in *Corynebacterium glutamicum*

Considering the potential toxicity to cell viability posed by the accumulation of ribulose 1,5-bisphosphate (R15P), a product catalyzed by PRK (Zhao et al., 2024), we prioritized the introduction of a heterologous RuBisCO enzyme that catalyzes the subsequent step in the metabolic pathway before incorporating PRK. Previous studies have demonstrated the successful heterologous expression of the RuBisCO protein, composed of CbbL and CbbS subunits derived from *H*. *neapolitanus* (Heinhorst et al., 2006; Baumgart et al., 2017), in *C. glutamicum*, underscoring the feasibility of our research approach.

**FIGURE 3:**
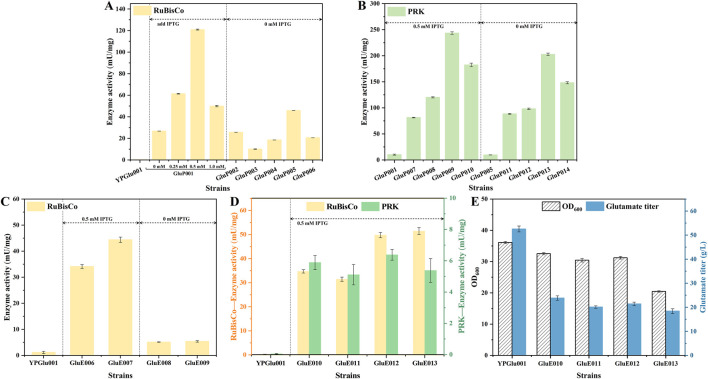
Optimized expression of RuBisCO and PRK. **(A)** Selection of promoters to enhance RuBisCO expression based on the plasmid pXMJ19. **(B)** Selection of promoters to enhance PRK expression based on the plasmid pZ8. **(C)** Enzyme activity assays of RuBisCO expressed from the genome under P_tac_ or P_H30_ promoters. **(D)** Enzyme activity assays of RuBisCO and PRK expressed from the genome. **(E)** Shake-flask fermentation evaluation of RuBisCO pathway-engineered strains and parental control. Enzyme activities are expressed as mU per mg of total soluble protein in crude extracts to reflect the relative enzyme loading in each strain. Data points in A to E are presented as mean ± SD from three independent biological replicates.

To achieve coordinated expression of RuBisCO and PRK, we first optimized and screened promoters for the *cbbLS* gene encoding RuBisCO. Considering the “detoxification” function of RuBisCO, a series of relatively strong promoters were selected to regulate its expression. Specifically, the inducible P_tac_ promoter and several constitutive promoters with varying strengths, including P_tuf_, P_pgk_, P_H5_, P_H30_, and P_H36_, were employed. Among these constitutive promoters, P_tuf_ and P_pgk_ are recognized as strong promoters (Wei et al., 2018; Henke et al., 2021), while the synthetic H-series promoters show increasing strengths in the order of P_H5_, P_H30_, and P_H36_(Yim et al., 2013). Each promoter was used to drive *cbbLS* expression on the plasmid pXMJ19, resulting in the construction of six plasmids: pXMJ19-P_tac_-*cbbLS*, pXMJ19-P_tuf_-*cbbLS*, pXMJ19-P_pgk_-*cbbLS*, pXMJ19-P_H5_-*cbbLS*, pXMJ19-P_H30_-*cbbLS*, and pXMJ19-P_H36_-*cbbLS*. The recombinant plasmids were transformed into the parental strain *C. glutamicum* YPGlu001, generating six engineered strains (GluP001–GluP006). In GluP001, the target gene was driven by the IPTG inducible P_tac_ promoter, so we tested induction with 0, 0.25, 0.5, and 1.0 mM IPTG to compare the concentration effects on the expression of *cbbLS*. RuBisCO activities were measured in crude cell extracts and normalized to the total soluble protein content (mU/mg total protein) to reflect the enzyme’s relative abundance and functional contribution within the cellular protein pool (Figure 3A). Based on this metric, the inducible P_tac_ promoter (strain GluP001) and the constitutive P_H30_ promoter (strain GluP005) demonstrated the highest expression efficiencies, yielding significantly higher RuBisCO activities compared to the other candidates.

To determine the optimal RuBisCO–PRK combination for enhancing glutamate conversion rates, we selected two RuBisCO expression backgrounds (inducible P_tac_ and constitutive P_H30_) for overexpression of the PRK gene, which was derived from *M. hungatei* based on our prior research, and further screened PRK promoters to identify the most suitable expression levels. Specifically, the *prk* was expressed on the expression vector pZ8 using strong promoters P_tac_ and P_sod_ (Henke et al., 2021), the medium-strength promoter P_fba_ (Yan et al., 2022), and the thermoregulatory promoter P_groES_ (Cheng et al., 2019), resulting in the construction of plasmids pZ8-P_tac_-PRK, pZ8-P_sod_-PRK, pZ8-P_fba_-PRK and pZ8-P_groES_-PRK. These recombinant plasmids were then transformed into the strain GluP001 and GluP005, yielding strains GluP007 (GluP001, pZ8-P_tac_-prk), GluP008 (GluP001, pZ8-P_sod_-prk), GluP009 (GluP001, pZ8-P_fba_-prk), GluP010 (GluP001, pZ8-P_groES_-prk), GluP011 (GluP005, pZ8-P_tac_-prk), GluP012 (GluP005, pZ8-P_sod_-prk), GluP013 (GluP005, pZ8-P_fba_-prk), and GluP014 (GluP005, pZ8-P_groES_-prk). PRK activity in these engineered strains was measured by enzymatic assays, with the results shown in Figure 3B. The *prk* gene, controlled by the P_fba_ and P_groES_ promoters, showed high activity in both RuBisCO bearing strains controlled by the inducible P_tac_ and the constitutive P_H30_, respectively. Notably, PRK activity was significantly higher in strain GluP001 with the inducible expression of RuBisCO than in GluP005 with the constitutive expression of RuBisCO.

Next, to eliminate the metabolic burden imposed by plasmids and enhance the stability of gene expression, we integrated RuBisCO and PRK into the genome of the strain. We specifically targeted intergenic regions as integration sites to ensure stable expression of the target genes while minimizing potential disruptions to the host’s growth and metabolic homeostasis. Building upon the chassis strain in which glycolysis and the PPP pathway were blocked, and based on the results shown in Figure 3A, we further introduced the heterologous RuBisCO-based CO_2_ fixation pathway driven by either the P_tac_ or P_H30_ promoter into the genomes of GluE004 and GluE005, thereby generating four new strains: GluE006 (GluE004, Δ(BBD29_05350-BBD29_05355) :: (P_tac_-*cbbLS*)), GluE007 (GluE005, Δ(BBD29_05350-BBD29_05355) :: (P_tac_-*cbbLS*)), GluE008 (GluE004, Δ(BBD29_05350-BBD29_05355) :: (P_H30_-*cbbLS*)), and GluE009 (GluE005, Δ(BBD29_05350-BBD29_05355) :: (P_H30_-*cbbLS*)). Enzymatic assays were subsequently performed to measure RuBisCO activities in these strains (GluE006-GluE009), and the results are illustrated in Figure 3C. Notably, the RuBisCO activities in GluE006 and GluE007 reached 34.1 mU/mg and 44.38 mU/mg, respectively, positioning them as the top two strains with the highest RuBisCO activities. Consequently, these two strains were chosen for the subsequent integration of the *prk* gene.

Based on the results shown in Figure 3B, we introduced *prk* driven by either the P_fba_ or P_groES_ promoter into the genomes of GluE006 and GluE007, thereby generating four new strains: GluE010 (GluE006, Δ(BBD29_04380-BBD29_04385) :: (P_fba_-*prk*)), GluE011 (GluE006, Δ(BBD29_04380-BBD29_04385) :: (P_groES_-*prk*)), GluE012 (GluE007, Δ(BBD29_04380-BBD29_04385) :: (P_fba_-*prk*)), and GluE013 (GluE007, Δ(BBD29_04380-BBD29_04385) :: (P_groES_-*prk*)). These strains, together with the parental strain YPGlu001, were subjected to enzymatic assays to evaluate the expression of RuBisCO and PRK, with the results shown in Figure 3D. Among all tested strains, GluE012 and GluE013, constructed using GluE005 as the chassis strain exhibited superior enzyme activities. Specifically, the GluE013 strain showed the highest RuBisCO activity (approximately 51.43 mU/mg), while the GluE012 strain exhibited the highest PRK activity (approximately 6.38 mU/mg). In comparison, GluE010 and GluE011, constructed using GluE004 as the parental strain, exhibited overall lower enzyme activities than GluE012 and GluE013. Among them, GluE010 demonstrated comparatively higher RuBisCO activity (approximately 34.69 mU/mg) and PRK activity (approximately 5.89 mU/mg). The observed functionalities of both enzymes provide compelling evidence for the successful establishment of a heterologous RuBisCO-mediated CO_2_ fixation pathway. Subsequently, we performed shake-flask fermentation tests on strains GluE010 to GluE013, using YPGlu001 as the control. The cell growth and glutamate production results at 48 h are shown in Figure 3E. It is noteworthy that the experimental strains did not achieve the expected performance. Among all the strains, the control strain YPGlu001 still exhibited the best performance, with an OD_600_ of 36.1 and a glutamate titer of 52.56 g/L. Among the four experimental strains, GluE010 relatively showed the best performance. It reached an OD_600_ of 32.54 and a glutamate titer of 23.86 g/L. These values represent respective decreases of 9.86% and 54.6%, compared with YPGlu001. The results indicated that substituting a portion of the central carbon metabolism with the heterologous RuBisCO pathway was ineffective, suggesting that the RuBisCO pathway did not fully compensate for the functions of glycolysis and the PPP. This limitation might be addressed through adaptive laboratory evolution (ALE).

### 3.3 Adaptive laboratory evolution under CO_2_ stress for improved RuBisCO-dependent growth

Adaptive laboratory evolution (ALE) has demonstrated its efficacy as a powerful method for enhancing cellular adaptation to adverse environments. In this study, despite the introduction of a heterologous pathway provided functional enzymes for catalysis, the resulting efficiency was notably inadequate. Consequently, ALE of the engineered strains under CO_2_ stress was employed to enhance the activity of the heterologous RuBisCO pathway.

**FIGURE 4:**
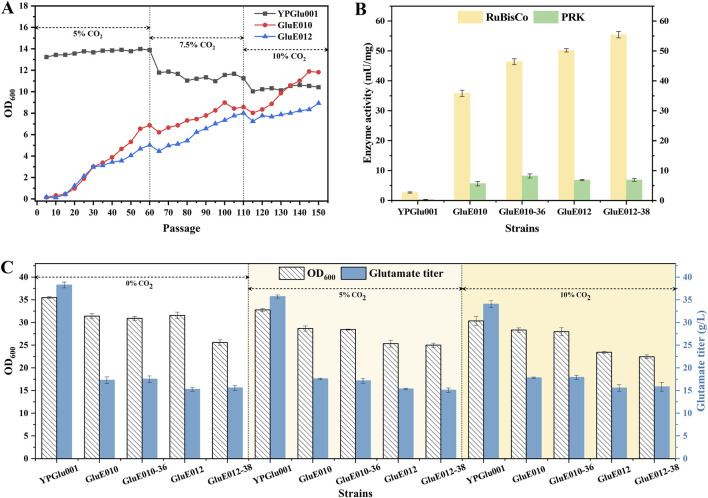
Adaptive evolution of strains GluE010 and GluE012 to elevated CO_2_ concentrations, and enzyme activities and production performance of the evolved strains. **(A)** Cell growth of YPGlu001, GluE010, and GluE012 strains after 150 generations of continuous passage at 5%, 7.5%, and 10% CO_2_ concentrations. **(B)** Expression levels of RuBisCO and PRK in representative evolved strains. **(C)** Shaking flask fermentation of representative evolved strains was performed under different CO_2_ concentrations. Data points in B to C are presented as mean ± SD from three independent biological replicates.

Based on the two blocking strategies employed for the glycolysis in this study, we obtained two background strains, and on this basis, the RuBisCO gene regulated by the tac promoter and the PRK gene regulated by different promoters were integrated into the genome. Subsequently, one strain with superior enzyme activity as well as relatively better cell growth and glutamate production performance was selected from each background chassis. GluE010 and GluE012 were chosen for adaptive laboratory evolution (ALE). Specifically, during cultivation, the CO_2_ concentration was gradually increased from 5% to 10%. Concurrently, Strain YPGlu001 served as a control, subjected to identical evolutionary selection pressure. Specifically, cultures were passaged for 60 generations at 5% CO_2_, then transferred to 7.5% CO_2_ for 50 generations, and finally maintained at 10% CO_2_ for 40 generations, totaling 150 generations. Three parental strains underwent laboratory adaptive evolution for 150 generations to obtain evolved strains. Serial passages were carried out with the criterion that the OD_600_ value should reach approximately 3 ensuring viable cell growth throughout the entire passage process. Following each passage, the remaining cell culture was allowed to grow further until it attained a plateau OD_600_ value, enabling an assessment of their growth capacity. The OD_600_ plateau values, recorded every five passages, are depicted in Figure 4A. As shown in the figure, cell growth was initially inhibited at each increased CO_2_ concentration. Growth gradually recovered after several passages. Ultimately, after 150 generations of adaptive evolution, GluE010 reached an OD_600_ of 11.82, while GluE012 reached an OD_600_ of 8.93. The bacterial suspension was evenly spread on plate slants and incubated overnight, yielding multiple single colonies. Several strains were then randomly selected from these colonies for verification, including GluE010-36 and GluE012-38, which were used for subsequent experiments.

A comparative analysis was conducted to assess the activities of RuBisCO and PRK in the evolved GluE010-36 and GluE012-38 strains versus their parental counterparts, GluE010 and GluE012, with the outcomes presented in Figure 4B. Specifically, Figure 4B demonstrates that the RuBisCO activity in GluE010-36 and GluE012-38 increased by 29.5% and 10.4%, respectively, when compared to the non-evolved strains GluE010 and GluE012. Meanwhile, the PRK activity in GluE010-36 increased by 45.1%, compared to the non-evolved strain GluE010. The minor activity detected in the YPGlu001 strain was considered as the baseline background noise of the spectrophotometric assay. Nevertheless, during the course of adaptive evolution, the PRK activity in the GluE012-38 strain failed to show any improvement (Figure 4B). Comparative sequence analysis of the RuBisCO-encoding genes in the evolved GluE010-36 and GluE012-38 strains revealed no mutations. This indicates that the enhanced RuBisCO activity did not result from sequence alterations at the gene level. Furthermore, we performed genomic sequencing on the evolved strains GluE010-36 and GluE012-38, with results presented in the Supplementary Material (Supplementary Table S2). Although the RuBisCO-related genes did not mutate, there were 57 common mutations across the genome of the evolved strains, covering many processes including transcriptional regulation, the oxidoreductase system, the central carbon metabolism, etc. Notably, there were still 11 uniquely mutated genes in strain GluE012-38. The above results indicate that adaptive evolution exerted a profound impact on the genome of the strains, which benefits cell growth and viability under CO_2_ conditions; however, unraveling the precise underlying mechanisms necessitates further investigation.

Further, we conducted shake-flask fermentations of the strains obtained after adaptive evolution with the starting strains as controls to evaluate the combined effects of pathway engineering and adaptive evolution on cell growth and glutamate production. Unfortunately, as shown in Figure 4C, although adaptive evolution enhanced enzyme activity, it did not confer beneficial effects on either growth or production. Under 0%, 5%, and 10% CO_2_ conditions, all engineered strains exhibited lower growth and glutamate production than the control strain YPGlu001. Unlike the recovery of growth observed during serial transfers in ALE under CGXII medium (Figure 4A), no improvement in growth was evident during shake-flask fermentation in nutrient-rich medium. On the contrary, as illustrated by the fermentation of GluE012 under the 0% CO_2_ condition, cell growth even decreased by 18.89% after adaptive evolution. These results suggest that genetic perturbation of the glycolysis and PPP pathways may disrupt central metabolism and greatly hamper cell growth. Notably, with increasing CO_2_ concentration, glutamate production in YPGlu001 gradually declined, whereas the engineered strains maintained relatively stable production across the three tested conditions, which may be attributable to the introduction of the heterologous RuBisCO-mediated CO_2_ fixation pathway.

### 3.4 Combination of RuBisCO-based CO_2_ fixation and the central carbon metabolism for improved glutamate yield

**FIGURE 5:**
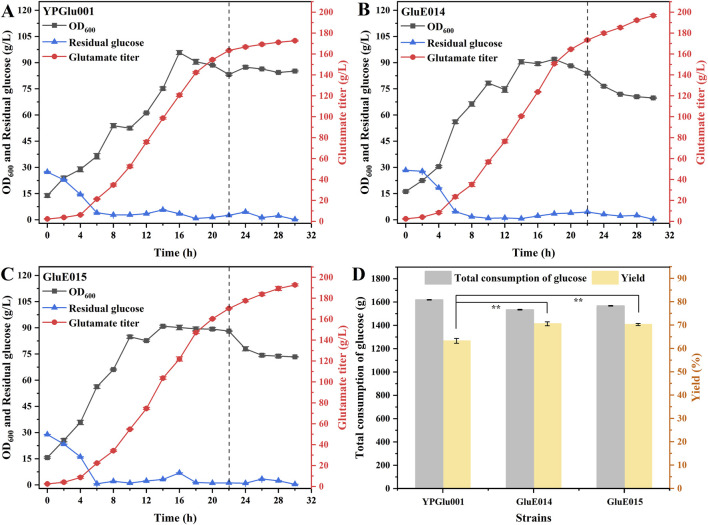
The influence of RuBisCO-PRK pathway introduction on the fermentation performance of strains in a 5 L bioreactor. **(A)** Fermentation process curve of YPGlu001 in a 5 L bioreactor over 30 h. **(B)** Fermentation process curve of GluE014 in a 5 L bioreactor over 30 h. **(C)** Fermentation process curve of GluE015 in a 5 L bioreactor over 30 h. **(D)** Total glucose consumed and product yield of the strains after 30 h of fermentation. Data points in A to D are presented as mean ± SD from three independent biological replicates. Statistical significance is indicated as * for P < 0.05, ** for P < 0.01, and “ns” represents no significance.

In the aforementioned pursuit, the obstruction of glycolysis and the PPP metabolism could significantly disrupt cellular metabolic equilibrium, ultimately impeding the optimal functioning of the RuBisCO pathway. Rather than relying solely on an alternative metabolic route, an alternative attempt was made to integrate RuBisCO complementation with the central carbon metabolism. Guided by the preceding optimization efforts focused on the promoters for *cbbLS* and *prk*, the *tac* promoter was strategically selected to regulate the expression of *cbbLS*. Concurrently, two promoters, P_groES_ and P_fba_, were chosen to govern the expression of *prk*. Consequently, the strain designated as GluE014 (*C. glutamicum* YPGlu001, Δ(BBD29_05350-BBD29_05355):: (P_tac_-*cbbLS*), Δ(BBD29_04380-BBD29_04385):: (P_groES_-*prk*)), and strain GluE015 (*C. glutamicum* YPGlu001, Δ(BBD29_05350-BBD29_05355):: (P_tac_-*cbbLS*), Δ(BBD29_04380-BBD29_04385):: (P_fba_-*prk*)) were constructed. Fed-batch fermentations of strains GluE014, GluE015, and the control strain *C. glutamicum* YPGlu001 were carried out in a 5 L bioreactor for 30 h, and the results are shown in Figure 5.

In terms of cell growth, as shown in Figure 5, GluE014 and GluE015 entered exponential and stationary phases earlier than the control strain YPGlu001. During the exponential phase, both engineered strains exhibited markedly enhanced cell growth, and their biomass remained higher than that of the control strain throughout the stationary phase. Specifically, at 14 h, GluE014 and GluE015 reached the highest biomass (OD_600_ of 90.9) upon entering the stationary phase. Their biomass also remained consistently higher than that of YPGlu001 across the exponential phase, with the most pronounced difference observed at 10 h, when the biomass of GluE014 and GluE015 exceeded that of YPGlu001 by 49.14% and 61.71%, respectively. This improvement was most likely attributable to the incorporation of the RuBisCO pathway, which effectively promoted central carbon metabolism. In contrast, YPGlu001 entered the stationary phase at around 20 h and remained in this phase until the end of fermentation. Notably, beginning at around 22 h, the OD_600_ values of GluE014 and GluE015 showed a marked decline. For instance, the biomass of GluE015 decreased from about 88 to about 78 before stabilizing at a new equilibrium around OD_600_ 73. In comparison, YPGlu001 maintained relatively stable growth, with OD_600_ values remaining around 86. This phenomenon may reflect a metabolic rebalancing process in the late fermentation stage, during which intracellular resources were redistributed from growth toward glutamate production. Consequently, fermentation entered a new phase characterized by reduced biomass but enhanced production.

In terms of glutamate production, all three strains showed similar accumulation rates during the early and middle stages of fermentation. However, corresponding to the trends in cell growth, starting from around 22 h, YPGlu001 entered the stationary phase, and its glutamate production rate began to slow down significantly. Between 22 h and 30 h, only 9.26 g/L of glutamate was accumulated, corresponding to a production rate of 1.16 g/L/h. In contrast, glutamate productivities in strains GluE014 and GluE015 were significantly higher than in YPGlu001 after 22 h of fermentation. From 22 h to 30 h, GluE014 accumulated an additional 26.55 g/L of glutamate, corresponding to a production rate of 3.32 g/L/h, 1.86-fold higher than that of YPGlu001. During the same period, GluE015 accumulated 22.53 g/L of glutamate, with a production rate of 2.82 g/L/h, 1.43-fold higher than that of YPGlu001. To further compare the glutamate production, the yield of glutamate was calculated. Compared to the consumption of added glucose, the utilizable carbon from corn syrup was negligible (Supplementary Table S3). Additionally, due to batch-to-batch variations in reducing sugar content, corn syrup was excluded from our analysis to ensure consistent comparisons. As evidenced by the yield from glucose, the integration of the complementary RuBisCO pathway in strains GluE014 and GluE015 resulted in a notable enhancement in glutamate production. Specifically, after 30 h of fed-batch fermentation, YPGlu001 produced 172.71 g/L of glutamate, whereas GluE014 and GluE015 produced 196.78 g/L and 192.76 g/L of glutamate, representing increases of 13.94% and 11.61%, respectively, compared with the control strain. Furthermore, both engineered strains consumed less glucose during fermentation than the control strain. Specifically, GluE014 exhibited a total glucose consumption of 1534.23 g, representing a 5.24% reduction relative to YPGlu001, GluE015 consumed 1567.34 g of glucose, 3.20% lower than YPGlu001. Finally, regarding glutamate yield from glucose, YPGlu001 achieved 63.23%, while GluE014 and GluE015 demonstrated 70.53% and 70.23%, respectively, corresponding to relative improvements of 11.55% and 11.07% (Figure 5D). During late-stage fermentation, GluE014 and GluE015 exhibited efficient glutamate production coupled with relatively suppressed cell growth, suggesting an optimized balance between biomass accumulation and glutamate synthesis. Collectively, these results demonstrate that CO_2_, as a supplementary carbon source to glucose, effectively reconstructed carbon metabolism and enhanced the synthesis of the target product.

## 4 Discussion

In the microbial production of glutamate from glucose, the theoretical yield is 81.7%. However, in practice, the yield is typically only 69%–71%, accompanied by CO_2_ release, which causes carbon loss and environmental pollution. The introduction of a RuBisCO-based CO_2_ fixation pathway as a supplementary route to central carbon metabolism has been proven to improve carbon utilization efficiency and promote target product synthesis (Pang et al., 2020; Rin Kim et al., 2022; van Aalst et al., 2022; Ting and Ng, 2023; van Aalst et al., 2023; Ben Nissan et al., 2024; Zhou et al., 2025). Therefore, in this study, to further improve the glutamate yield, we attempted to alter the normal glucose metabolism of the strain. A heterologous RuBisCO pathway was introduced to link glycolysis with the PPP, thereby rewiring the carbon metabolism of *C. glutamicum*. Using this strategy, the glutamate yield increased from 60.23% to 70.53%, representing an improvement of 11.55%; however, it still remains below the theoretical yield of 81.7%, indicating that further optimization is required.

Specifically, to achieve the redirection of metabolic flux, we knocked out the key genes *gap*, *gapX*, or *pgk* in glycolysis, as well as *zwf* in PPP, to block the native pathways and force carbon flux from F6P and G3P into PPP. On this basis, *prk* and *cbbLS* were introduced to construct the heterologous RuBisCO pathway, which converts ribose-5-phosphate (R5P) into 3-PG and reconstructs glucose metabolism. In addition, we screened promoters for *prk* and *cbbLS* expression, and adopted the *tac* promoter for *cbbLS* expression, while the *groES* and *fba* promoters were employed for *prk* expression. During this process, we found that the disruption of GAPDH or PGK expression did not affect cell growth, probably because PPP partially compensated for glycolysis and maintained cellular metabolism. However, knockout of *zwf* caused a significant decrease in growth and amplified the negative effects of GAPDH or PGK deletion. The knockout of *pgk* exerted a greater impact compared to *gap* and *gapX* deletion, which may be related to the accumulation of 1,3-BGP. Since 1,3-BGP cannot be metabolized through other pathways, its accumulation may lead to waste of cellular energy or reducing power. In contrast, G3P can be metabolized through PPP, and therefore the effect of blocking its downstream metabolism on cell growth was relatively smaller.

Subsequently, the heterologous RuBisCO pathway was integrated into the genome of YPGlu001. To improve the catalytic efficiency of the functional enzymes, ALE under different CO_2_ concentrations was carried out, which enhanced both strain growth and enzyme activity. However, during shake-flask fermentation, the engineered strains exhibited much lower growth and production compared with the parental strain YPGlu001. Similar “replacement” strategies have also been applied in the synthesis of other products. For example, Zhou et al. reported that knockout of the *zwf* and *pfk* genes blocked the oxidative PPP and glycolysis, while the RuBisCO pathway was introduced for succinate production, with xylose serving as the carbon source for the supply of ribulose-5-P. Compared with glucose as a substrate, xylose appeared to be a more efficient precursor for ribulose-5-P, yet the succinate yield remained unsatisfactory. They proposed that this limitation might be due to insufficient ATP and reducing power in the reconstructed pathway (Zhou et al., 2025). In our study, the replacement of glycolysis and PPP with the RuBisCO pathway likewise failed to improve glutamate production, which might also be attributed to the same reason, since the heterologous RuBisCO pathway requires additional ATP, thereby exacerbating the energy burden of the cells. Therefore, to make such a “replacement” strategy effective, future metabolic engineering should focus on enhancing ATP and reducing power supply. In contrast to the direct knockout strategy, Ng et al. attenuated *zwf* and *pfkAB* expression via CRISPR interference (CRISPRi) to facilitate RuBisCO-based CO_2_ fixation for the production of 5-aminolevulinic acid (5-ALA). In *E. coli* Nissle, they successfully synchronized the CO_2_ fixation pathway with central metabolism by fine-tuning the heterologous pathway through CRISPRi, achieving efficient 5-ALA synthesis from xylose and reducing CO_2_ release by 77% (Effendi and Ng, 2024). This study highlights that balancing the heterologous RuBisCO pathway with the native central carbon metabolism is crucial for efficient production of target compounds. In our study, the complete blockage of glycolysis and the PPP may have led to insufficient supply of key intermediates, while the heterologous RuBisCO pathway could not fully substitute the metabolic functions of glycolysis and PPP. In summary, the replacement of the native glucose metabolism with the heterologous RuBisCO pathway did not achieve the expected improvement in glutamate conversion. In addition to optimizing the supply of ATP and reducing power, attenuating rather than completely abolishing the endogenous glucose metabolism may represent a more effective strategy.

Considering that the blockage of glucose metabolic pathways disrupts cellular metabolic balance, in our study, the *tac* promoter obtained from promoter optimization was used to regulate the *cbbLS* gene, while the *groES* and *fba* promoters were used to regulate the *prk* gene. The heterologous RuBisCO pathway was directly integrated into the genome of the parental strain YPGlu001 as a supplement to the strain’s carbon metabolism. The resulting engineered strains, GluE014 and GluE015, both showed improved glutamate production and conversion rates. Among them, the strain with the highest performance, GluE014, exhibited a 13.94% increase in glutamate titer and an 11.61% improvement in conversion yield compared with YPGlu001. Notably, during fermentation, GluE014 and GluE015 entered the exponential, stationary, and decline phases earlier than YPGlu001, indicating that the introduction of the heterologous RuBisCO pathway effectively promoted the metabolic process of the strains. The heterologous RuBisCO pathway reintroduced CO_2_ into the central metabolic pathways, which improved carbon utilization efficiency, indirectly reduced the dependence on exogenous glucose, and promoted glutamate accumulation.

In addition, regarding promoter selection, the 5 L fermenter results showed that overexpression of PRK in GluE015 did not lead to higher glutamate production. Among the selected *groES* and *fba* promoters, although PRK activity under the *fba* promoter was higher than under the *groES* promoter, the corresponding strain exhibited reduced glutamate production during fermentation. As depicted in Supplementary Figure S1, the measurement of CO_2_ levels in the off-gas reveals that strains GluE014 and GluE015 exhibited significantly reduced CO_2_ emissions compared to YPGlu001, thereby underscoring the functional efficacy of the RuBisCO module. Notably, during the late-stage fermentation, GluE015 demonstrated even lower CO_2_ levels than GluE014, a finding that aligns with the utilization of a stronger promoter in GluE015. The above results suggest that excessive PRK expression likely disrupted the metabolic equilibrium between the heterologous pathway and host physiology. In summary, a moderate level of PRK expression is essential to maintain the balance between the heterologous pathway and the host metabolism.

Taken together, the RuBisCO cycle, as a supplement to carbon metabolism, improves carbon utilization efficiency through CO_2_ fixation, and enhances glutamate production and conversion efficiency. At the same time, moderately controlling PRK expression is crucial for preventing intermediate accumulation, maintaining metabolic balance, and achieving increased product yield.

## 5 Conclusion

In this study, a heterologous RuBisCO pathway was introduced into a high-glutamate-producing strain, aiming to maximize CO_2_ utilization for enhancing yield. A comprehensive comparison was conducted between two distinct strategies: one involving the block of the glycolysis and the PPP metabolism and the alternative construction of the RuBisCO pathway, and the other focusing on the combination of RuBisCO with the native central carbon metabolism. Notably, the combination strategy yielded a positive impact on glutamate yield enhancement, underscoring the promising potential of harnessing CO_2_ for industrial chemical production.

## Data Availability

The original contributions presented in the study are included in the article/Supplementary Material, further inquiries can be directed to the corresponding author.
