# Supplementary material for: RuBisCO-based CO2 fixation improves glutamate production in Corynebacterium glutamicum
Source: Front Bioeng Biotechnol. 2026 Feb 27;14:1783749. doi: 10.3389/fbioe.2026.1783749 (PMC12982323; doi:10.3389/fbioe.2026.1783749)
Supplement: Supplementary file 1 [file Supplementaryfile1.docx]

***Supplementary Material***

**RuBisCO-based CO_2_ fixation improves glutamate production in *Corynebacterium glutamicum***

Aiying Wei^1, 2†^, Jingui Liu^1†^, Yulin Tang^1^, Gang Meng^2^, Chunguang Zhao^2^, Houbo Su^2^, Heyun Wu^1, 3^, Qian Ma^1, 3^, Xixian Xie^1, 3*^

^1^College of Biotechnology, Tianjin University of Science and Technology, Tianjin, 300457, China

^2^Ningxia Eppen Biotech Co., Ltd., Yinchuan, 750100, China

^3^Key Laboratory of Industrial Fermentation Microbiology, Ministry of Education, Tianjin University of Science and Technology, Tianjin, 300457, China

^†^These authors contributed equally to this work and share first authorship.

*Correspondence:

Xixian Xie

xixianxie@tust.edu.cn


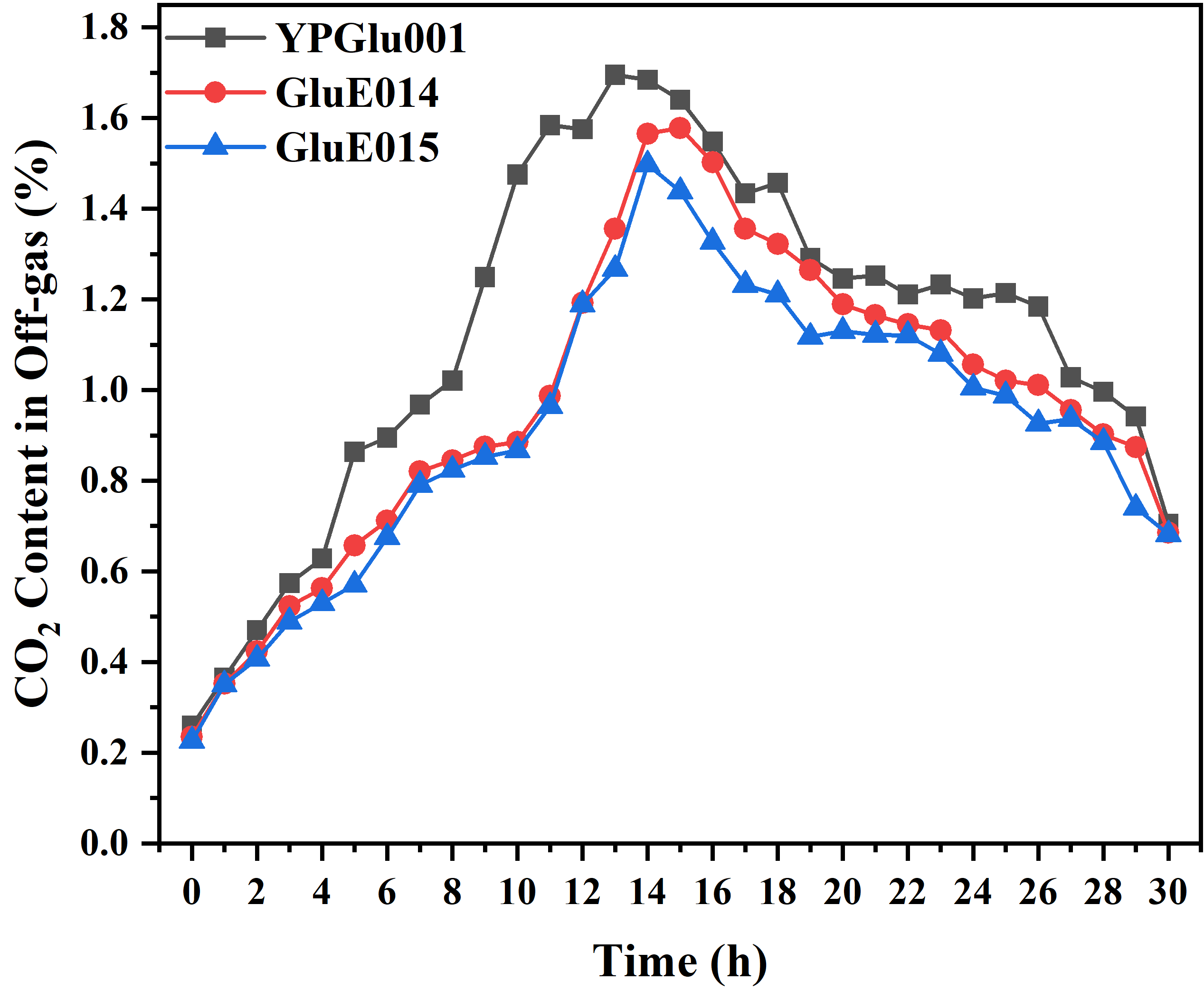


**Supplementary Figure S1** Dynamic profiles of off-gas CO_2_ concentration in 5 L bioreactor.


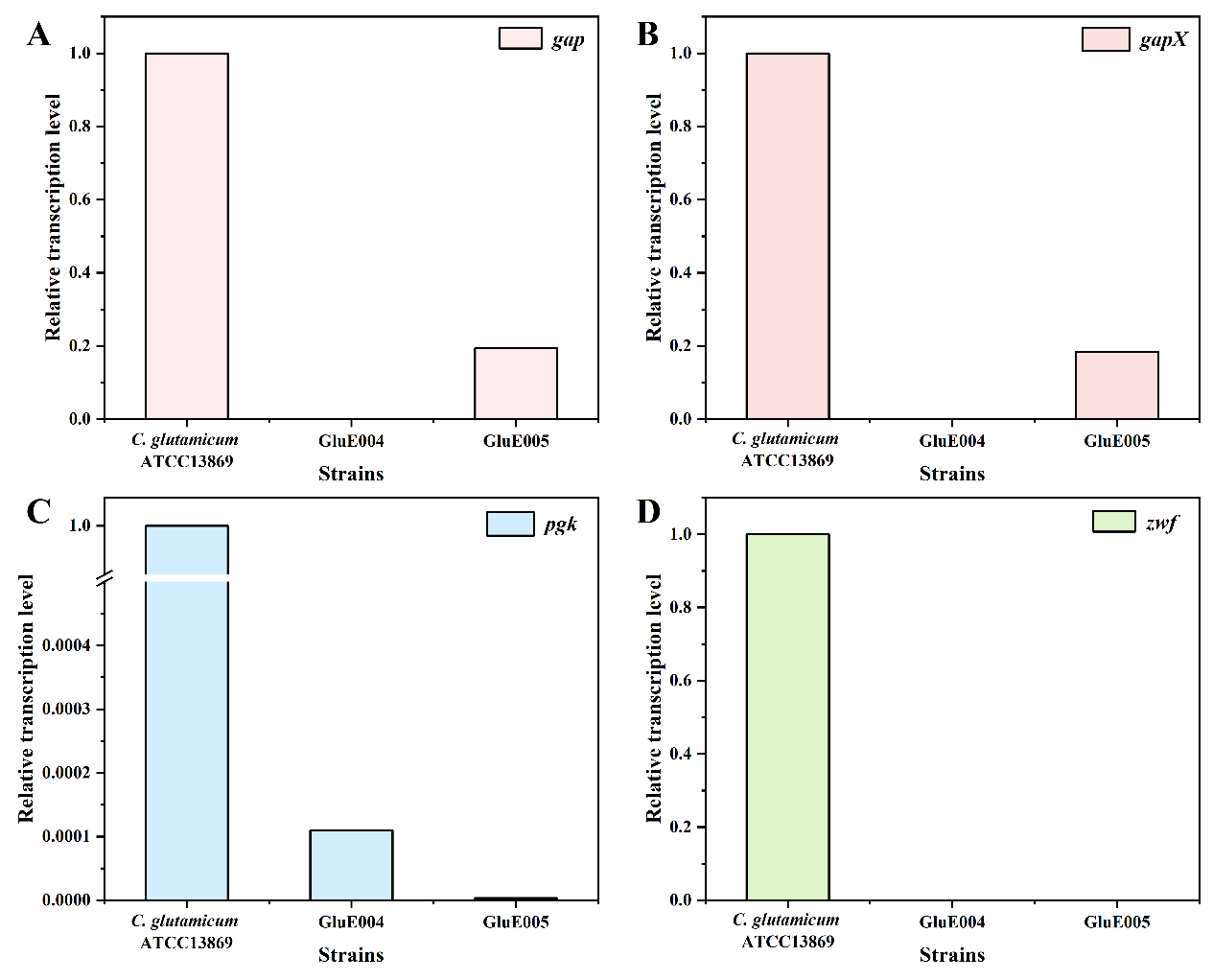


**Supplementary Figure S2** Relative transcription levels of *gap*, *gapX*, *pgk*, and *zwf*.

**Supplementary Table S1** Primers used in this study.

| Name | Sequence (5′–3′) |
| --- | --- |
| pK18mobsacB-F | TCCTGTGTGAAATTGTTATCCGCT |
| pK18mobsacB-R | GTCGTGACTGGGAAAACCCTGGCG |
| Δ*gap*-up-F | GGGTAACGCCAGGGTTTTCCCAGTCACGACCACCAAAGCCGTCAGAGACGAATG |
| Δ*gap*-up-R | TGTTTAAGTTTAGTGGATGGGCTGCGTCTGACCGAGCTCGTAG |
| Δ*gap*-down-F | CCCATCCACTAAACTTAAACAGACCAACACGAATGGTCATGTTG |
| Δ*gap*-down-R | ATTGTGAGCGGATAACAATTTCACACAGGAGGCTGATCCTCAAATGACCAAG |
| Δ*gap*-Verification-F | TGGTAGGGAGGCAATGATTC |
| Δ*gap*-Verification-R | CCCCGCTAAAGTTGAGGAC |
| Δ*gapX*-up-F | GGGTAACGCCAGGGTTTTCCCAGTCACGACATTGTTGTCCTCGGCGTTTC |
| Δ*gapX*-up-R | GTGTTCAGGGTCAAGTTGAGCGTTGTTGTTGCGGTGTAGG |
| Δ*gapX*-down-F | CCTACACCGCAACAACAACGCTCAACTTGACCCTGAACAC |
| Δ*gapX*-down-R | ATTGTGAGCGGATAACAATTTCACACAGGAGGAAGCTCAGCATGGGTATC |
| Δ*gapX*-Verification-F | TCGATTGCGAAGGGTAGTGC |
| Δ*gapX*-Verification-R | TTCATGCCCAGCGGATTGTG |
| Δ*zwf*-up-F | GGGTAACGCCAGGGTTTTCCCAGTCACGACCTCGTTGCTTCTTGGAGCGA |
| Δ*zwf*-up-R | ATTTCATCGGTTGAGCTCGTGTGCGG |
| Δ*zwf*-down-F | GCTCAACCGATGAAATGCTTTCCCGCA |
| Δ*zwf*-down-R | ATTGTGAGCGGATAACAATTTCACACAGGACTGCAGCCAAATCCACCG |
| Δ*zwf*-Verification-F | TCGTTGCTTCTTGGAGCGA |
| Δ*zwf*-Verification-R | CTGCAGCCAAATCCACCG |
| Δ*pgk*-up1-F | GGGTAACGCCAGGGTTTTCCCAGTCACGACGCAAAGGTCCTGAACGACAAG |
| Δ*pgk*-up1-R | CCCATCCACTAAACTTAAACAGAGGGTCTTAACAGCCATGC |
| Δ*pgk*-down1-F | TGTTTAAGTTTAGTGGATGGGAAGGAACTCCCAGGCGTTGC |
| Δ*pgk*-down1-R | ATTGTGAGCGGATAACAATTTCACACAGGAAGTGCCGATAGCCCACACTG |
| Δ*pgk*-Verification1-F | TGACCAAGGTCTCCGGCAAC |
| Δ*pgk*-Verification1-R | CCAGGTTCATCTTCCAGTTACC |
| Δ*pgk*-Internal1-F | CAACGATGACCGCGAGATCAC |
| Δ*pgk*-Internal1-R | GAGAATTGCAACGCCTGGGAG |
| Δ*pgk*-up2-F | GGGTAACGCCAGGGTTTTCCCAGTCACGACGACAACGAGTGGGGCTAC |
| Δ*pgk*-up2-R | GAGTTCCTTGCCTAGGTTTCGCGTGGGTCGAAG |
| Δ*pgk*-down2-F | CACGCGAAACCTAGGCAAGGAACTCCCAGGC |
| Δ*pgk*-down2-R | ATTGTGAGCGGATAACAATTTCACACAGGAAGTGCCGATAGCCCACACTG |
| P_tac_-*cbbLS-*UP-F | GGGTAACGCCAGGGTTTTCCCAGTCACGACGGGCTCGGAATGATCTTGAC |
| P_tac_-*cbbLS-*UP-R | CTTTCGCGGTATGGCATGATAGCCAAAGAGCCCTCCACAAC |
| P_tac_-*cbbLS-*F | GGTACCCAGCTTTTGCCAAAGAGCCCTCCACAAC |
| P_tac_-*cbbLS-*R | CGTATGGCAATGACAGTTTGAGTTAGTTGCCGCGGTAGACC |
| P_tac_-*cbbLS-*down-F | GGTCTACCGCGGCAACTAACTCAAACTGTCATTGCCATACG |
| P_tac_-*cbbLS-*down-R | ATTGTGAGCGGATAACAATTTCACACAGGACTCCAAATGGTTCCGTTATCTC |
| P_H30_-*cbbLS-*UP-F | GGGTAACGCCAGGGTTTTCCCAGTCACGACGGGCTCGGAATGATCTTGAC |
| P_H30_-*cbbLS-*UP-R | GGTACCCAGCTTTTGCCAAAGAGCCCTCCACAAC |
| P_H30_-*cbbLS-*F | GTTGTGGAGGGCTCTTTGGCAAAAGCTGGGTACCAAAGT |
| P_H30_-*cbbLS-*R | CGTATGGCAATGACAGTTTGAGTTAGTTGCCGCGGTAGACC |
| P_H30_-*cbbLS-*down-F | GGTCTACCGCGGCAACTAACTCAAACTGTCATTGCCATACG |
| P_H30_-*cbbLS-*down-R | ATTGTGAGCGGATAACAATTTCACACAGGACTCCAAATGGTTCCGTTATCTC |
| P_fba_-*prk-*UP-F | GGGTAACGCCAGGGTTTTCCCAGTCACGACCCGTTCGGCTGACTCCTTCT |
| P_fba_-*prk-*UP-R | GCACTATACTTTTTAACTGCCCCAACGTCATCAAAAAATCCGC |
| P_fba_-*prk-*F | GCGGATTTTTTGATGACGTTGGGGCAGTTAAAAAGTATAGTGC |
| P_fba_-*prk-*R | CCGCATCCAAACTCACTTAGTCCTTTGGTCGAAAAAAAAAGCCCGCACTGTCAGGTGCGGGCTTTTTTC |
| P_fba_-*prk-*down-F | GAAAAAAGCCCGCACCTGACAGTGCGGGCTTTTTTTTTCGACCAAAGGACTAAGTGAGTTTGGATGCGG |
| P_fba_-*prk-*down-R | ATTGTGAGCGGATAACAATTTCACACAGGACTCACTAGTACGCGGATAAATG |
| P_groES_-*prk-*UP-F | GGGTAACGCCAGGGTTTTCCCAGTCACGACCCGTTCGGCTGACTCCTTCT |
| P_groES_-*prk-*UP-R | AATTTCATCCAACGTCATCAAAAAATCCGCCGTTCCT |
| P_groES_-*prk-*F | GCGGATTTTTTGATGACGTTGGATGAAATTGAGCGAATTAAG |
| P_groES_-*prk-*R | CCGCATCCAAACTCACTTAGTCCTTTGGTCGAAAAAAAAAGCCCGCACTGTCAGGTGCGGGCTTTTTTC |
| P_groES_-*prk-*down-F | GAAAAAAGCCCGCACCTGACAGTGCGGGCTTTTTTTTTCGACCAAAGGACTAAGTGAGTTTGGATGCGG |
| P_groES_-*prk-*down-R | ATTGTGAGCGGATAACAATTTCACACAGGACTCACTAGTACGCGGATAAATG |
| pXMJ19-P_tac_-F1 | TAAGCTTGCATGCCTGCAGAAGGAGATATACATATGGCAG |
| pXMJ19-P_tac_-R1 | TCGAGCTCGGTACCCGGGTTAGTTGCCGCGGTAGACCTTAAAAAGTATAGTGCTG |
| pXMJ19-P_tuf_-F1 | GAAAATCCTGTTTGATGGTGGTTTTATTGCTGAGCGCAACGGCAC |
| pXMJ19-P_tuf_-R1 | CTGCCATATGTATATCTCCTTCTTCGTGGTGGCTACGACTTTCG |
| pXMJ19-P_pgk_-F1 | GAAAATCCTGTTTGATGGTGGTTACACCTGCCAGCTCCTGC |
| pXMJ19-P_pgk_-R1 | CTGCCATATGTATATCTCCTTCGATTGTTTAAAAAATTCTTCTGGTGCAA |
| pXMJ19-P_H5_-F1 | GAAAATCCTGTTTGATGGTGGTTCAAAAGCTGGGTACCGGTGGTCGTGCTGACTCTACGGGGGAGGAAGTTCAGCTGGTACTGCTCGCGTTGGGCTGATAAAGGAGTAGAGTTGGATCCATGGCAGTTAAAAAGTATAGTGC |
| pXMJ19-P_H5_-R1 | GCACTATACTTTTTAACTGCCATGGATCCAACTCTACTCCTTTATCAGCCCAACGCGAGCAGTACCAGCTGAACTTCCTCCCCCGTAGAGTCAGCACGACCACCGGTACCCAGCTTTTGAACCACCATCAAACAGGATTTTC |
| pXMJ19-P_H30_-F1 | GAAAATCCTGTTTGATGGTGGTTCAAAAGCTGGGTACCAAAGTAACTTTTCGGTTAAGGTAGCGCATTCGTGGTGTTGCCCGTGGCCCGGTTGGTTGGGCAGGAGTATATTGGGATCCATGGCAGTTAAAAAGTATAGTGC |
| pXMJ19-P_H30_-R1 | GCACTATACTTTTTAACTGCCATGGATCCCAATATACTCCTGCCCAACCAACCGGGCCACGGGCAACACCACGAATGCGCTACCTTAACCGAAAAGTTACTTTGGTACCCAGCTTTTGAACCACCATCAAACAGGATTTTC |
| pXMJ19-P_H36_-F1 | GAAAATCCTGTTTGATGGTGGTTCAAAAGCTGGGTACCTCTATCTGGTGCCCTAAACGGGGGAATATTAACGGGCCCAGGGTGGTCGCACCTTGGTTGGTAGGAGTAGCATGGGATCCATGGCAGTTAAAAAGTATAGTGC |
| pXMJ19-P_H36_-R1 | GCACTATACTTTTTAACTGCCATGGATCCCATGCTACTCCTACCAACCAAGGTGCGACCACCCTGGGCCCGTTAATATTCCCCCGTTTAGGGCACCAGATAGAGGTACCCAGCTTTTGAACCACCATCAAACAGGATTTTC |
| pXMJ19-SeqPrimer-R | AAGCGGTCCACGTGGTTTGC |
| pXMJ19-SeqPrimer-F | CAGAATCTTCAACCGGCACATG |
| pXMJ19-GenPrimer-R | GAAAATCCTGTTTGATGGTGGTTTTATTGCTGAGCGCAACGGCAC |
| pXMJ19-GenPrimer-F | TGAAGCCGAACACGTTACCAACC |
| pZ8-P_tac_-F1 | GGATTTTTTGATGctgtgcaggtcgtaaatcactgc |
| pZ8-P_tac_-R1 | CTGCCATATGTATATCTCCTTCGATTGTTTAAAAAATTCTTCTGGTGCAA |
| pZ8-P_sod_-F1 | GGCAGCAGATCAATTCGTAGCTGCCAATTATTCCGGGCTTG |
| pZ8-P_sod_-R1 | GAAATTTTCAGGCTGACTCATATGTATATCTCCTTCGGTTTCCGCACCGAGCATATAC |
| pZ8-P_fba_-F1 | GAAAATCCTGTTTGATGGTGGTTCAAAAGCTGGGTACCAAAGTAACTTTTCGGTTAAGGTAGCGCATTCGTGGTGTTGCCCGTGGCCCGGTTGGTTGGGCAGGAGTATATTGGGATCCATGGCAGTTAAAAAGTATAGTGC |
| pZ8-P_fba_-R1 | GAAATTTTCAGGCTGACTCATATGTATATCTCCTTCTGACTGTCACTGAACTATGTATGTAATTTC |
| pZ8-P_groES_-F1 | GGCAGCAGATCAATTCGACGTTGGATGAAATTGAGCGAATTAAGG |
| pZ8-P_groES_-R1 | GCACTATACTTTTTAACTGCCATGGATCCCATGCTACTCCTACCAACCAAGGTGCGACCACCCTGGGCCCGTTAATATTCCCCCGTTTAGGGCACCAGATAGAGGTACCCAGCTTTTGAACCACCATCAAACAGGATTTTC |
| pZ8-SeqPrimer-R | CTGCGTTCTGATTTAATCTGTATCAG |
| pZ8-SeqPrimer-F | GGAGCTGCATGTGTCAGAGG |
| pZ8-GenPrimer-R | GCTGTTGACAATTAATCATCGGCTC |

**Supplementary Table S2** Genomic mutations in evolved strains GluE010-36 and GluE012-38.

| No. | Gene | Function | Substitution | Strains |
| --- | --- | --- | --- | --- |
| 1 | BBD29_00820 | hypothetical protein | D187N | GluE010-36, GluE012-38 |
| 2 | BBD29_01230 | NAD(P)H-quinone oxidoreductase | G49R | GluE010-36, GluE012-38 |
| 3 | BBD29_01275 | molybdenum cofactor biosynthesis protein | G33D | GluE010-36, GluE012-38 |
| 4 | BBD29_01465 | hypothetical protein | D977N | GluE010-36, GluE012-38 |
| 5 | BBD29_01485 | surface-anchored protein | P220L | GluE010-36, GluE012-38 |
| 6 | BBD29_01700 | Crp/Fnr family transcriptional regulator | E2K | GluE010-36, GluE012-38 |
| 7 | *sdhA* | succinate dehydrogenase flavoprotein subunit | R133C | GluE012-38 |
| 8 | *sdhA* | succinate dehydrogenase flavoprotein subunit | P301S | GluE012-38 |
| 9 | BBD29_02520 | 4-hydroxyphenylpyruvate dioxygenase | G409R | GluE010-36, GluE012-38 |
| 10 | BBD29_02555 | iron ABC transporter permease | A177T | GluE010-36, GluE012-38 |
| 11 | BBD29_02750 | 2-succinyl-5-enolpyruvyl-6-hydroxy-3-cyclohexene-1-carboxylic-acid synthase | D335N | GluE010-36, GluE012-38 |
| 12 | BBD29_03895 | 2-methylcitrate dehydratase | A144T | GluE010-36, GluE012-38 |
| 13 | BBD29_03940 | hypothetical protein | A63V | GluE010-36, GluE012-38 |
| 14 | BBD29_04005 | thioesterase | E195K | GluE010-36, GluE012-38 |
| 15 | BBD29_04045 | ABC transporter permease | T320I | GluE010-36, GluE012-38 |
| 16 | BBD29_04090 | helicase | R256* | GluE010-36, GluE012-38 |
| 17 | BBD29_04095 | restriction endonuclease subunit M | A24V | GluE010-36, GluE012-38 |
| 18 | BBD29_04165 | thymidylate kinase | A158T | GluE010-36, GluE012-38 |
| 19 | BBD29_04295 | ATP-dependent DNA helicase | A324V | GluE010-36, GluE012-38 |
| 20 | BBD29_04585 | rRNA methyltransferase | P54S | GluE010-36, GluE012-38 |
| 21 | BBD29_04835 | DNA-binding response regulator | R4L | GluE010-36, GluE012-38 |
| 22 | BBD29_04875 | molybdopterin molybdenumtransferase | T279I | GluE010-36, GluE012-38 |
| 23 | BBD29_04905 | alpha-amylase | V45A | GluE010-36, GluE012-38 |
| 24 | BBD29_04985 | transcriptional regulator | P108A | GluE012-38 |
| 25 | BBD29_04990 | cadmium transporter | A2V | GluE012-38 |
| 26 | BBD29_04990 | cadmium transporter | D94N | GluE012-38 |
| 27 | BBD29_05105 | urea ABC transporter substrate-binding protein | A21C | GluE012-38 |
| 28 | BBD29_05195 | two-component sensor histidine kinase | G64S | GluE010-36, GluE012-38 |
| 29 | *proY* | proline-specific permease ProY | G194V | GluE010-36, GluE012-38 |
| 30 | BBD29_06325 | homoserine dehydrogenase | D404G | GluE012-38 |
| 31 | BBD29_06395 | hypothetical protein | G71D | GluE010-36, GluE012-38 |
| 32 | BBD29_06405 | molybdopterin molybdenumtransferase MoeA | H180N | GluE010-36, GluE012-38 |
| 33 | BBD29_06410 | cyclic pyranopterin phosphate synthase | G73S | GluE010-36, GluE012-38 |
| 34 | BBD29_06415 | long-chain fatty acid--CoA ligase | G32S | GluE010-36, GluE012-38 |
| 35 | BBD29_06955 | restriction endonuclease subunit R | T839I | GluE010-36, GluE012-38 |
| 36 | *rihB* | ribonucleoside hydrolase | A163V | GluE010-36, GluE012-38 |
| 37 | BBD29_08445 | transketolase | T237I | GluE010-36, GluE012-38 |
| 38 | BBD29_08925 | integrase catalytic subunit | P91S | GluE012-38 |
| 39 | BBD29_09105 | toxin repressor | G149D | GluE010-36, GluE012-38 |
| 40 | BBD29_09340 | tellurium resistance protein TerC | G127D | GluE010-36, GluE012-38 |
| 41 | BBD29_09715 | RNA-binding transcriptional accessory protein | G198D | GluE012-38 |
| 42 | BBD29_09720 | LacI family transcriptional regulator | G288R | GluE010-36, GluE012-38 |
| 43 | BBD29_10200 | isoleucine--tRNA ligase | I794T | GluE010-36, GluE012-38 |
| 44 | BBD29_10465 | adenosylcobinamide-GDP ribazoletransferase | L100F | GluE010-36, GluE012-38 |
| 45 | BBD29_10580 | bifunctional glutamine-synthetase adenylyltransferase/deadenyltransferase | A364T | GluE010-36, GluE012-38 |
| 46 | BBD29_10625 | bifunctional RNase H/acid phosphatase | D179N | GluE010-36, GluE012-38 |
| 47 | BBD29_11085 | ribokinase | L128F | GluE010-36, GluE012-38 |
| 48 | BBD29_11095 | Na^+^-dependent transporter | I26V | GluE010-36, GluE012-38 |
| 49 | BBD29_11410 | acetyl-CoA acetyltransferase | T377A | GluE010-36, GluE012-38 |
| 50 | BBD29_11485 | LuxR family transcriptional regulator | E25K | GluE010-36, GluE012-38 |
| 51 | BBD29_11595 | alkylhydroperoxidase | W231R | GluE010-36, GluE012-38 |
| 52 | BBD29_11600 | ABC transporter ATP-binding protein | S310F | GluE010-36, GluE012-38 |
| 53 | BBD29_11900 | gluconate kinase | L104P | GluE012-38 |
| 54 | BBD29_12535 | acetyl-CoA hydrolase | S372C | GluE010-36, GluE012-38 |
| 55 | BBD29_12720 | dethiobiotin synthase | G42D | GluE010-36, GluE012-38 |
| 56 | BBD29_12850 | trehalose-phosphatase | V69I | GluE010-36, GluE012-38 |
| 57 | BBD29_12880 | FAD-binding dehydrogenase | T157I | GluE010-36, GluE012-38 |
| 58 | BBD29_13195 | spermidine synthase | A396T | GluE010-36, GluE012-38 |
| 59 | BBD29_13385 | acetyltransferase | V174M | GluE010-36, GluE012-38 |
| 60 | BBD29_13395 | cardiolipin synthase A | V174I | GluE010-36, GluE012-38 |
| 61 | BBD29_13460 | pyridine nucleotide-disulfide oxidoreductase | A200T | GluE010-36, GluE012-38 |
| 62 | BBD29_13530 | hypothetical protein | S24L | GluE012-38 |
| 63 | BBD29_13615 | 2-polyprenylphenol hydroxylase | G65E | GluE010-36, GluE012-38 |
| 64 | BBD29_13780 | ABC transporter permease | W49* | GluE010-36, GluE012-38 |
| 65 | BBD29_13865 | 3-methyladenine DNA glycosylase | A155T | GluE010-36, GluE012-38 |
| 66 | BBD29_13990 | SAM-dependent methyltransferase | P192S | GluE010-36, GluE012-38 |
| 67 | BBD29_14000 | tellurite resistance protein | S170F | GluE010-36, GluE012-38 |
| 68 | BBD29_14835 | inositol 1-phosphate synthase | A319V | GluE010-36, GluE012-38 |

**Supplementary Table S3** The contents of glucose, reducing sugar and total sugar in three batches of corn syrup.

| Batches | Glucose (g/L) | Reducing sugar (g/L) | Total sugar (g/L) |
| --- | --- | --- | --- |
| 1 | 2.2 | 26.6 | 34.6 |
| 2 | 2.5 | 30.7 | 40.5 |
| 3 | 2.3 | 27.5 | 37.4 |

**Supplementary gene sequences**

**RuBisCO gene from *Halothiobacillus neapolitanus***

*cbbL*

ATGGCAGTTAAAAAGTATAGTGCTGGTGTAAAAGAATACCGGCAGACCTATTGGATGCCGGAATACACACCGTTGGATTCCGACATCCTTGCATGCTTCAAAATCACCCCACAACCGGGTGTTGATCGCGAAGAAGCCGCAGCCGCGGTTGCAGCAGAATCTTCAACCGGCACATGGACCACCGTGTGGACCGATTTGCTGACCGACATGGACTACTACAAAGGCCGTGCCTATCGCATTGAAGACGTACCCGGTGACGATGCGGCATTCTATGCCTTTATCGCCTACCCAATCGACCTGTTCGAAGAAGGGTCAGTTGTTAACGTGTTTACCTCACTGGTTGGTAACGTGTTCGGCTTCAAAGCGGTACGCGGCCTGCGTCTGGAAGATGTTCGCTTCCCACTCGCCTACGTTAAAACCTGTGGCGGCCCACCGCACGGTATTCAAGTCGAACGCGACAAGATGAACAAATATGGTCGCCCACTGTTGGGTTGCACCATCAAGCCAAAACTTGGTTTGTCTGCGAAAAACTACGGCCGTGCCGTATACGAGTGCCTCCGTGGCGGCCTCGACTTCACTAAAGATGATGAAAACATCAATTCTCAGCCGTTCATGCGCTGGCGCGATCGCTTCTTGTTCGTACAAGACGCGACCGAAACTGCTGAAGCCCAAACCGGCGAACGCAAAGGCCATTACCTCAACGTAACGGCGCCAACTCCTGAAGAAATGTACAAGCGCGCAGAATTCGCCAAAGAAATTGGCGCGCCAATCATTATGCACGACTACATCACCGGTGGCTTCACGGCCAACACTGGCTTGGCCAAGTGGTGTCAAGACAACGGCGTACTGCTGCACATCCACCGTGCGATGCATGCGGTTATCGACCGTAACCCGAACCACGGTATTCACTTCCGTGTTCTGACCAAGATTCTGCGTTTGTCGGGTGGCGATCACCTGCACACCGGTACCGTTGTCGGCAAACTGGAAGGCGACCGTGCCTCTACTCTGGGCTGGATTGATTTGCTCCGCGAATCGTTTATCCCTGAAGATCGCTCGCGCGGTATCTTCTTCGATCAAGACTGGGGTTCAATGCCAGGCGTATTCGCTGTGGCCTCTGGTGGTATTCACGTATGGCACATGCCTGCGCTGGTTAACATCTTTGGTGACGACTCTGTCCTCCAATTCGGTGGCGGTACGCTGGGTCATCCATGGGGCAACGCTGCCGGTGCTGCTGCCAACCGTGTTGCTCTGGAAGCCTGCGTAGAAGCGCGTAACCAAGGCCGCGATATCGAAAAAGAAGGCAAAGAAATTCTGACTGCTGCTGCACAGCACAGCCCAGAACTGAAGATTGCCATGGAAACTTGGAAAGAGATCAAATTCGAATTTGACACTGTCGACAAACTCGACACTCAAAATCGTTGA

*cbbS*

ATGGCTGAAATGCAGGATTACAAGCAAAGCCTCAAATATGAGACTTTCTCTTATCTTCCACCCATGAACGCGGAACGCATCCGCGCTCAAATCAAGTACGCAATTGCTCAAGGCTGGAGCCCCGGCATTGAGCACGTAGAAGTGAAAAACTCCATGAACCAATATTGGTACATGTGGAAACTTCCCTTCTTCGGCGAACAAAATGTCGACAACGTGTTGGCTGAAATTGAAGCGTGTCGTAGTGCGTATCCAACACACCAGGTCAAACTGGTGGCTTATGACAACTATGCGCAAAGCTTAGGTCTGGCCTTCGTGGTCTACCGCGGCAACTAA

**PRK gene from *Methanospirillum hungatei***

ATGAGTCAGCCTGAAAATTTCCGGGAAGTAATTCGGCATTCTCCATTGGTCTATCTTATTGGAGTCGCAGGAGATTCCGGTTCAGGTAAATCAACTTTTACACGAGCAATATCAGATATTTTCGGTGAAGAACTCGTCTCTTCTATCACGGTAGATGATTACCATCTCTATGACAGAAAAACACGAAGTGAGATGGGTATAACCCCTCTGCTCCATACAGCAAATAATCTGAAATTATTGGAAGAAAATCTGATGGACCTGAAGGCAGGCAGAACAATACAAAAACCGGTATACCTGCATGATCATGGAACCTTTGGTGAACCTGAACTATTCTCTCCGACGAAATTTATCATAATCGAAGGACTACATCCATACGCAACGAAATCTCTTCGCGCGCTTTATGATTACACCATCTTTGTGGATCCGGAACGAGATGTTAAATATGACTGGAAGATACGGCGGGATATGAAGAAACGAAACTACGATAAAAATGAAGTTCTTCGTGAAATTCTTCAGCGTGAACCAGATTATTTTCAATATGTGTTCCCCCAAAGGGAGGTGGCTGATGCAGTCATCCAGATTTCTTACTCATCGTATGGAAAGGAAGAAGGAGAGAAACGCAATGTATACCGTGTTATGCTCTCGATGCCGGCACAAGAATACTGCTTTGAAGATATTGAATTGAATATCGACCTGTGTGACTTATTTAAAAAATCATCCCATGATTTCTCATTATCCTGTATCTCCCATACTCCGGATTCACGAAATATGCGTGCTCTTGTTGTGGATGGAGAACTGATGCCAGACACCATCCATAAGATAGAACGGCAGATAGAATTCCAGACAGGTATATCACCAATTAATATTTTCAGAGGTCAGGAACATATCACCGGAACCGACCTTGTACGGCTGATACTCTCCTGGCAGATAATCAATGGAAGAATAGCATTATCCAATCATCTGGATCAATAA
